# Supplementary material for: Exome sequencing and analysis of 454,787 UK Biobank participants
Source: Nature. 2021 Oct 18;599(7886):628–34. doi: 10.1038/s41586-021-04103-z (PMC8596853; doi:10.1038/s41586-021-04103-z)
Supplement: Supplementary file 1 — This file contains the Supplementary Discussion, Supplementary Note (including a list of investigators from the Regeneron Genetics Center and a list of investigators from the DiscovEHR cohort), Supplementary Figures 1–10 and the Supplementary References. [file 41586_2021_4103_MOESM1_ESM.pdf]

nature portfolio

https://doi.org/10.1038/s41586-021-04103-z

Supplementary information

Exome sequencing and analysis of 454,787 UK Biobank participants

In the format provided by the authors and unedited

Exome sequencing and analysis of 454,787 UK Biobank participants

SUPPLEMENTARY INFORMATION

Joshua D. Backman[1], Alexander H. Li[1], Anthony Marcketta[1], Dylan Sun[1], Joelle Mbatchou[1], Christopher E. Gillies[1], Daren Liu[1], Adam Locke[1], Suganthi Balasubramanian[1], Ashish Yadav[1], Nila Banerjee[1], Michael D. Kessler[1], Amy Damask[1], Simon Liu[1], Christian Benner[1], Xiaodong Bai[1], Evan Maxwell[1], Lauren Gurski[1], Kyoko Watanabe[1], Jack A. Kosmicki[1], Veera Rajagopal[1], Jason Mighty[1], Regeneron Genetics Center[2], DiscovEHR[3], Marcus Jones[1], Lyndon Mitnaul[1], Giovanni Coppola[1], Eric Jorgenson[1], Lucas Habegger[1], William J. Salerno[1], Alan R. Shuldiner[1], Luca A. Lotta[1], John D. Overton[1], Michael N. Cantor[1], Jeffrey G. Reid[1], George Yancopoulos[1], Hyun M. Kang[1], Jonathan Marchini[1]†, Aris Baras[1]†, Gonçalo R. Abecasis[1*]†, Manuel A. Ferreira[1*]†

SUPPLEMENTARY DISCUSSION

Limitations and caveats

The following caveats should be considered when interpreting results from our study.

A small number of associations were observed with variants flagged to have low quality. Of the 12 million coding variants identified, 447,533 (3.7%) were flagged as potentially having low quality by our machine learning approach. However, of these variants, only 53 were found among our top 8,865 variant-trait association pairs discovered in the UKB cohort, including 14 pairs for which no other variant in the same gene provided stronger evidence for association (index variant in **Supplementary Table 6**). Six of these variant-trait pairs were tested for replication in the DiscovEHR cohort, of which four (67%) were found to have a significant and consistent association. As such, we conclude that only a small number of potentially low-quality variants were among the top associations and, for most of these, the observation that the trait associations replicated in an independent cohort indicates that genotyping errors (if present) did not lead to false-positive associations.

Associations explained by correlated traits. We found a few examples of associations with a given trait A that were likely explained by a stronger association with a correlated trait B. This is analogous to the rare vs. common variant confounding effect that we addressed systematically through formal conditional analysis, but in this case involving correlated traits (instead of variants). For example, we noticed that rare variants in *SLC27A3* – which we found to be associated with lower asthma risk (**Supplementary Table 7**) – also had a sub-threshold association with higher

lung function, specifically forced expiratory volume in 1 second (FEV_1; effect = 0.037 s.d. units, 95% CI 0.015 to 0.058, *P*=0.0009). However, the same variant had a stronger association with height (effect = 0.054 s.d. units, 95% CI 0.039 to 0.070, $P=10^{-11}$), which is highly correlated with FEV_1. When we tested the association between *SLC27A3* and FEV_1 after controlling for height, the association was no longer significant (effect = 0.006 s.d. units, 95% CI -0.012 to 0.024, *P*=0.513). Another example of this was sepsis (specifically the diagnosis code ICD10 A41), for which we found four genes with rare variant associations with higher disease risk: three genes with somatic mutations found in patients with myeloid leukemia (ML) and other hematological malignancies (*ASXL1*, *JAK2*, *SRSF2*), and one gene related to chronic kidney disease (*PKD1*). Individuals with ML have a high risk of sepsis[1], and so we hypothesized that the associations between sepsis and *ASXL1*, *JAK2* and *SRSF2* were at least partly confounded by disease status for ML. Consistent with this possibility, the association between sepsis and all three genes was greatly attenuated after controlling for ML status (*ASXL1*: $P=3\text{x}10^{-4}$; *JAK2*: $P=2\text{x}10^{-6}$; *SRSF2*: *P*=0.002). Similarly, *PKD1* has a very strong association with kidney disease (for example, a burden of singleton pLOF variants increases risk of cystic kidney disease by 450-fold; **Supplementary Data 2**) and, in turn, kidney disease is a risk factor for sepsis[2]. When we controlled for cystic kidney disease, the association between sepsis and *PKD1* decreased from an odds ratio of 9.8 (95% CI 5 to 19, $P=2\text{x}10^{-11}$; **Supplementary Data 2**) to an odds ratio of 3.9 (95% 1.9 to 7.9, *P*=0.0001), suggesting that the association between *PKD1* and sepsis is at least partly explained by a confounding effect of kidney disease. Overall, these findings suggest that traits that increase risk of sepsis (such as ML and kidney disease) are likely to explain the rare variant associations with sepsis observed in this study. It is likely that there are other examples of trait associations that are explained by correlated traits. Unlike the analogous effect of common variant associations

sometimes explaining rare variant associations, which can be controlled for systematically by first identifying and then adjusting for common variant signals through conditional analysis, it is not straightforward to identify and control for the impact of correlated traits at scale. Instead, ad-hoc trait-specific analyses may be more appropriate to understand in greater detail associations such as those described above.

Burden tests when the effects of rare variants are in different directions. The burden tests we performed were not designed to identify associations with genes that harbor both trait-increasing and trait-lowering rare variants, and are expected to provide limited power in these instances. Other approaches have been developed for these situations, such as SKAT[3]/SKAT-O[4].

Enrichment of rare variant associations near GWAS signals. For this analysis, we considered only the subset of GWAS sentinel variants (identified by approximate conditional analysis using GCTA-COJO[5]) that were located >10Mb apart. We did this to ensure that a given gene could only be mapped to a single GWAS sentinel variant (except for the widest gene-set tested: all genes within 10Mb of a sentinel variant). However, by doing so, genes located near any additional independent signals located <10Mb apart did not contribute to the gene-sets tested (e.g. nearest gene to a GWAS sentinel variant), but instead were included among the genes that defined the control gene-set (i.e. rest of the genome). If genes located near these additional peaks are enriched for significant rare variant associations, this would tend to attenuate the overall enrichment estimated in our analysis. This could potentially explain the lower enrichment observed for quantitative traits (see **Supplementary Notes** and **Supplementary Figure 10a**), which had a greater number of GWAS sentinel variants overall (often located <10Mb apart) when compared to

binary traits (**Extended Data Fig 4b**). Our analysis also did not control for differences in gene size (and related variables, such as number of heterozygous carriers) between genes in the test set (e.g. nearest gene to a GWAS sentinel variant) and genes in the rest of the genome. We repeated the analysis using Firth regression, for each trait testing the association between significance of the gene burden test (significant vs. not significant) and location relative to GWAS sentinel variants (e.g. nearest gene vs. not nearest gene), while controlling for gene size, and results were largely unchanged (not shown). We also note that a recent exome-wide association study of six lipid phenotypes in 170,000 individuals also found that genes located in GWAS loci were enriched for significant rare variant associations, when compared to a set of genes located elsewhere in the genome and matched for gene size and total number of variants, among other variables[6]. For example, Hindy et al.[6] found that genes nearest to non-protein altering GWAS variants for HDL cholesterol were 4.1-fold more likely to have a rare variant association at $P<0.005$ when compared to a matched gene set. When considering GWAS loci that were explained by common coding variants, the enrichment was much larger (57-fold). However, a caveat of the analysis reported by Hindy et al. is that the associations with rare variants were estimated without conditioning on nearby common variant signals, which may explain some sub-threshold rare variant associations near GWAS loci. Nonetheless, the enrichment we observed among the nearest gene to GWAS sentinel variants in our analysis of HDL cholesterol (which included all GWAS loci discovered in UKB 450K TOPMed data, both with and without coding variants in LD with the sentinel variants) is consistent with that reported by Hindy et al.: 1.85-fold, 31.6-fold, 61.7-fold and 94.4-fold when using $P\leq0.05$, $P\leq10^{-4}$, $P\leq10^{-7}$ and $P\leq2.18\times10^{-11}$ to define significant gene burden associations.

No GWAS performed for 3,502 traits. Of the 3,994 traits tested for association with exome sequencing variants in individuals of European ancestry, 492 had at least one gene with a rare variant associated at $P \leq 2.18 \times 10^{-11}$. For these 492 traits (but not the remaining 3,502 traits) we performed a GWAS of variants imputed using the TOPMed reference panel, and used results from these analyses to subsequently (i) determine if rare variant associations from exome sequencing were independent of common variant signals from GWAS; and (ii) match quantitative traits with a relevant disease, through genetic correlation analyses. Because we did not run a GWAS for the remaining 3,502 traits, we (i) were not able to determine how many traits in total had common variant signals but no rare variant signals; and (ii) may have not matched quantitative traits with the most genetically correlated disease available among the 3,994 traits (only among the 492 traits with a GWAS).

Enrichment of FDA-approved targets among 564 genes associated with at least one trait. This analysis did not consider the possibility that the traits associated in the WES data with each of the 36 genes that are FDA-approved targets may be unrelated to the diseases for which the corresponding drug is approved for. Similarly, for some of the remaining 345 genes that are FDA-approved drug targets, we may not have tested any trait that is related to the approved indication.

SUPPLEMENTARY NOTES

Impact of burden test composition on yield of genetic associations

As noted, association of a phenotype with the burden of rare coding variants in a gene is a compelling way for human genetics to connect genes and disease[7]. We explored how often the 7,449 burden associations we discovered (for pLOF and/or deleterious missense variants with MAF≤1%) could be detected in single-variant analyses. On average across all genes, each burden test aggregated information from 299 rare variants, of which 37 were sufficiently common to be tested individually. Of the 7,449 burden associations discovered (**Supplementary Data 2**), 77.5% did not include any single variant with $P \leq 2.18 \times 10^{-11}$ in the set of aggregated variants. Relaxing the single-variant significance threshold to $P \leq 0.001$, we found that 1,791 (24.0%) burden associations had no individual variant associated *per se*, 2,198 (29.5%) had one associated variant, and 3,460 (46.4%) had two or more associated variants. These results show that burden associations are generally supported by multiple variants.

Next, we assessed the impact of allele frequency of individual variants on the yield of significant burden tests. For this analysis, we considered association results obtained after conditioning on common variant signals (details below) so as to minimize the potential confounding effect of LD between rare and common variants. Generally, burden tests that aggregated variants across a wider range of allele frequencies identified a larger number of significant associations overall. For example, when considering a burden of pLOF variants, we found 884 significant associations when aggregating information across variants with a MAF up to 1%, as compared to 500 associations for a MAF up to 0.001% (**Supplementary Table 16).** However, this was not because burden test associations were often explained by variants with a

greater MAF. Instead, we found that gene-trait associations often remained significant at $P \leq 2.18 \times 10^{-11}$ after excluding variants with a MAF between 0.1% and 1% from the burden test (**Extended Data Figure 6a)**. For example, 762 (86.2%) of the 884 associations with a burden of pLOFs discovered at MAF≤1% were also discovered at MAF≤0.1% (**Supplementary Table 16**). This pattern held as we focused the analysis on increasingly rarer variants, down to singletons. Therefore, the greater yield of associations with tests that aggregated variants with a MAF up to 1% is likely explained by the ability to capture in a single test association signals across a wide range of allele frequencies.

Finally, we compared the yield of associations between burden tests that considered only pLOF variants with those that included both pLOF and deleterious missense variants. When considering a burden of singletons, we found a total of 238 unique gene-trait associations across the two burden strategies, of which 136 (57.1%) were identified by both strategies, 56 (23.5%) only by pLOFs, and 46 (19.3%) only by aggregating pLOF and deleterious missense variants (**Extended Data Figure 6b**). At more permissive MAF thresholds, combining pLOFs and deleterious missense variants in the same test became progressively more valuable. For example, of 1,539 associations discovered by burden tests that included variants with a MAF≤1%, about half (655 or 42.6%) were discovered exclusively by aggregating pLOF and deleterious missense variants (**Extended Data Figure 6b**). These results demonstrate the utility of performing a variety of burden tests for discovery of genetic associations.

Enrichment of associations in GWAS loci

A major challenge for genetic association studies of complex traits is the identification of effector genes for the thousands of loci identified through GWAS, which often point to large numbers of variants in high LD and act through enhancers or other gene regulatory elements [8]. Identification of effector genes can require extensive *in vitro* experimental follow-up [9] and is especially challenging for GWAS loci that harbor many genes. We addressed the possibility that rare variant associations might help systematically pinpoint effector genes at GWAS loci.

We first determined how many of the 8,865 trait associations (across 564 genes and 492 traits) with rare coding variants discovered in Europeans were located within 1 Mb of a common variant GWAS signal for the same trait. To this end, we performed a GWAS for each of the 492 traits among individuals with WES data (see Methods, **Supplementary Data 1** and **Supplementary Figure 4**), identifying a total of 107,276 independent associations with common variants (hereafter "GWAS sentinel variants"). Of the 8,865 rare variant associations, 6,564 (74%) were within 1Mb of a GWAS sentinel variant for the same trait **(Extended Data Figure 4a**), when we expected only 3,736 (42%) to overlap by chance (see Methods). We then repeated the rare variant association analysis while adjusting for the GWAS common variant signals and found that most rare variant associations (8,059 of 8,865, 91%) remained significant at $P \leq 2.18 \times 10^{-11}$ (**Extended Data Figure 4c** and **Supplementary Data 2**). Thus, while rare variant signals and common variant signals were often near each other, they were almost always independent. However, we note that RV associations were more likely to be attenuated by GWAS signals if they included higher allele frequency variants, and if they were observed with single variants rather than burden tests (**Supplementary Table 17**).

We next proceeded to dissect the overlap between common GWAS signals and nearby rare variant signals in more detail. Specifically, for each trait, we compared the proportion of genes with a significant burden association ($P \leq 2.18 \times 10^{-11}$ after conditioning on GWAS sentinel variants) between genes located within 1 Mb of GWAS sentinel variants (considering only GWAS peaks located >10Mb apart) versus the remainder of the genome. Across all traits, we found that significant rare variant associations were 11.4-fold (95% CI 10.1 to 13.0, $P<10^{-300}$) more common in genes located within 1 Mb of a GWAS peak (**Figure 1**). Furthermore, we found that the signal for enrichment (i) decreased when we considered more liberal significance thresholds to define burden associations, dropping to 1.14-fold (95% CI 1.12 to 1.15) when using $P \leq 0.05$; (ii) progressively increased as we narrowed the window around each GWAS peak, reaching 59.4-fold (95% CI 51.8 to 68.2) when we focused only on the gene nearest to GWAS sentinel variants; and (iii) was stronger for binary (OR=61.3, 95% CI 40.3 to 93.2) than for quantitative (OR=10.4, 95% CI 9.1 to 11.9) traits (**Supplementary Figure 10a**). One caveat that could potentially explain the latter observation is that we restricted the enrichment analysis to GWAS signals located >10Mb apart; if genes located near any additional independent signals (which are more frequent for quantitative traits, due to higher power) are enriched for rare variant associations, this would tend to decrease the overall enrichment estimated in our analysis (see caveats in **Supplementary Discussion**). The diseases with largest enrichment of significant associations in genes nearest gene to GWAS peaks were hypothyroidism (288-fold) and type-2 diabetes (268-fold). Although the enrichment for quantitative traits was lower overall, some traits had a very large enrichment, such as vitamin D (809-fold), corneal hysteresis (707-fold) and airway obstruction (475-fold; **Supplementary Figure 10b**). These results show strong overlap between common variant signals

from GWAS and rare variant signals from exome-wide association studies, suggesting that rare variant burden signals will identify effector genes for thousands of GWAS loci.

Associations with brain imaging traits

In addition to the associations with *PLD1* discussed in the main text, other notable associations with brain imaging phenotypes include those between the iron transport gene transferrin (*TF*) and higher intra-cellular volume fraction (ICVF) derived from diffusion MRI, mostly with the cerebral peduncle and internal capsule; *GBE1* – which encodes a glycogen branching enzyme associated with adult polyglucosan body disease, an autosomal recessive leukodystrophy characterized by neurogenic bladder, progressive spastic gait, and peripheral neuropathy [10] – and both lower white matter lesion load and higher ICVF diffusion MRI measure; *PLEKHG3* – which encodes a protein that enhances polarized cell migration [11] – and diffusion MRI measures across several white matter tracts in a direction that reduces the magnitude of the primary axis diffusion (or increases dispersion); and associations between brain structures and *STAB1*, a scavenger receptor implicated in brain imaging phenotypes in the first 50K exome-sequenced individuals from the UKB [12], and that is located in a GWAS locus for bipolar disorder [13] (fifth nearest gene) and cognitive performance [14] (nearest gene).

List of investigators from the Regeneron Genetics Center

All authors are listed in alphabetical order.

RGC Management and Leadership Team

Goncalo Abecasis, D.Phil. [1], Aris Baras, M.D. [1], Michael Cantor, M.D. [1], Giovanni Coppola, M.D. [1], Andrew Deubler [1], Aris Economides, Ph.D. [1], Katia Karalis, Ph.D. [1], Luca A. Lotta, M.D., Ph.D. [1], John D. Overton, Ph.D. [1], Jeffrey G. Reid, Ph.D. [1], Katherine Siminovitch, M.D. [1], Alan Shuldiner, M.D. [1]

Contribution: All authors contributed to securing funding, study design and oversight. All authors reviewed the final version of the manuscript.

Sequencing and Lab Operations

Christina Beechert [1], Caitlin Forsythe, M.S. [1], Erin D. Fuller [1], Zhenhua Gu, M.S. [1], Michael Lattari [1], Alexander Lopez, M.S. [1], John D. Overton, Ph.D. [1], Maria Sotiropoulos Padilla, M.S. [1], Manasi Pradhan, M.S. [1], Kia Manoochehri, B.S. [1], Thomas D. Schleicher, M.S. [1], Louis Widom [1], Sarah E. Wolf, M.S. [1], Ricardo H. Ulloa, B.S. [1]

Contribution: Performed and are responsible for sample genotyping and exome sequencing, conceived and are responsible for laboratory automation, and responsible for sample tracking and the library information management system.

Clinical Informatics

Amelia Averitt, Ph.D. [1], Nilanjana Banerjee, Ph.D. [1], Michael Cantor, M.D. [1], Dadong Li, Ph.D. [1], Sameer Malhotra, M.D. [1], Deepika Sharma, MHI [1], Jeffrey Staples, Ph.D. [1]

Contribution: Development and validation of clinical phenotypes used to identify study participants and (when applicable) controls.

Genome Informatics

Xiaodong Bai, Ph.D. [1], Suganthi Balasubramanian, Ph.D. [1], Suying Bao, Ph.D. [1], Boris Boutkov, Ph.D. [1], Siying Chen, Ph.D. [1], Gisu Eom, B.S. [1], Lukas Habegger, Ph.D. [1], Alicia Hawes, B.S. [1], Shareef Khalid [1], Olga Krasheninina, M.S. [1], Rouel Lanche, B.S. [1], Adam J. Mansfield, B.A. [1], Evan K. Maxwell, Ph.D. [1], George Mitra, B.A. [1], Mona Nafde, M.S. [1], Sean O'Keeffe, Ph.D. [1], Max Orelus, B.B.A. [1], Razvan Panea, Ph.D. [1], Tommy Polanco, B.A. [1], Ayesha Rasool, M.S. [1], Jeffrey G. Reid, Ph.D. [1], William Salerno, Ph.D. [1], Jeffrey C. Staples, Ph.D. [1], Kathie Sun, Ph.D. [1], Jiwen Xin, Ph.D. [1]

Contribution: Performed and are responsible for analysis needed to produce exome and genotype data, provided compute infrastructure development and operational support, provided variant and gene annotations and their functional interpretation of variants, and conceived and are responsible for creating, developing, and deploying analysis platforms and computational methods for analyzing genomic data.

Analytical Genomics and Data Science

Goncalo Abecasis, D.Phil. [1], Joshua Backman, Ph.D. [1], Amy Damask, Ph.D. [1], Lee Dobbyn, Ph.D. [1], Manuel Allen Revez Ferreira, Ph.D. [1], Arkopravo Ghosh, M.S. [1], Christopher Gillies, Ph.D. [1], Lauren Gurski, B.S. [1], Eric Jorgenson, Ph.D. [1], Hyun Min Kang, Ph.D. [1], Michael Kessler, Ph.D. [1], Jack Kosmicki, Ph.D. [1], Alexander Li , Ph.D. [1], Nan Lin, Ph.D. [1], Daren Liu, M.S. [1], Adam Locke, Ph.D. [1], Jonathan Marchini, Ph.D. [1], Anthony Marcketta, M.S. [1], Joelle

Mbatchou, Ph.D. [1], Arden Moscati, Ph.D. [1], Charles Paulding, Ph.D. [1], Carlo Sidore, Ph.D. [1], Eli Stahl, Ph.D. [1], Kyoko Watanabe, Ph.D. [1], Bin Ye, Ph.D. [1], Blair Zhang, Ph.D. [1], Andrey Ziyatdinov, Ph.D. [1]

Contribution: Development of statistical analysis plans. QC of genotype and phenotype files and generation of analysis ready datasets. Development of statistical genetics pipelines and tools and use thereof in generation of the association results. QC, review and interpretation of result. Generation and formatting of results for manuscript figures.

Therapeutic Area Genetics

Ariane Ayer, B.S. [1], Aysegul Guvenek, Ph.D. [1], George Hindy, Ph.D. [1], Giovanni Coppola, M.D. [1], Jan Freudenberg, M.D. [1], Jonas Bovijn M.D. [1], Julie Horowitz, Ph.D. [1], Katherine Siminovitch, M.D. [1], Kavita Praveen, Ph.D. [1], Luca A. Lotta, M.D. [1], Manav Kapoor, Ph.D. [1], Mary Haas, Ph.D. [1], Moeen Riaz , Ph.D. [1], Niek Verweij, Ph.D. [1], Olukayode Sosina, Ph.D. [1], Parsa Akbari, Ph.D. [1], Priyanka Nakka, Ph.D. [1], Sahar Gelfman, Ph.D. [1], Sujit Gokhale, B.E. [1], Tanima De, Ph.D. [1], Veera Rajagopal, Ph.D. [1], Alan Shuldiner, M.D. [1], Bin Ye, Ph.D. [1], Gannie Tzoneva, Ph.D. [1], Juan Rodriguez-Flores, Ph.D. [1]

Contribution: Development of study design and analysis plans. Development and QC of phenotype definitions. QC, review, and interpretation of association results.

RGC Biology

Shek Man Chim, Ph.D. [1], Valerio Donato, Ph.D. [1], Aris Economides, Ph.D. [1], Daniel Fernandez, M.S. [1], Giusy Della Gatta, Ph.D. [1], Alessandro Di Gioia, Ph.D. [1], Kristen Howell, M.S. [1], Katia

Karalis, Ph.D. [1], Lori Khrimian, Ph.D. [1], Minhee Kim, Ph.D. [1], Hector Martinez [1], Lawrence Miloscio, B.S. [1], Sheilyn Nunez, B.S. [1], Elias Pavlopoulos, Ph.D. [1], Trikaldarshi Persaud, B.S. [1]

Contribution: Development of *in vivo* and *in vitro* experimental biology and interpretation.

Research Program Management & Strategic Initiatives

Esteban Chen, M.S. [1], Marcus B. Jones, Ph.D. [1], Michelle G. LeBlanc, Ph.D. [1], Jason Mighty, Ph.D. [1], Lyndon J. Mitnaul, Ph.D. [1], Nirupama Nishtala, Ph.D. [1], Nadia Rana, Ph.D. [1]

Contribution: Contributed to the management and coordination of all research activities, planning and execution, managed the review of the project.

Affiliations:

1. Regeneron Genetics Center, Tarrytown, NY USA

List of investigators from the DiscovEHR cohort

All authors are listed in alphabetical order.

Lance J. Adams[1], Jackie Blank[1], Dale Bodian[1], Derek Boris[1], Adam Buchanan[1], David J. Carey[1], Ryan D. Colonie[1], F. Daniel Davis[1], Dustin N. Hartzel[1], Melissa Kelly[1], H. Lester Kirchner[1], Joseph B. Leader[1], David H. Ledbetter, Ph.D.[1], J. Neil Manus[1], Christa L. Martin[1], Raghu P. Metpally[1], Michelle Meyer[1], Tooraj Mirshahi[1], Matthew Oetjens[1], Thomas Nate Person[1], Christopher Still[1], Natasha Strande[1], Amy Sturm[1], Jen Wagner[1], Marc Williams[1]

Affiliations:

1. Geisinger, Danville, PA, USA

SUPPLEMENTARY FIGURES

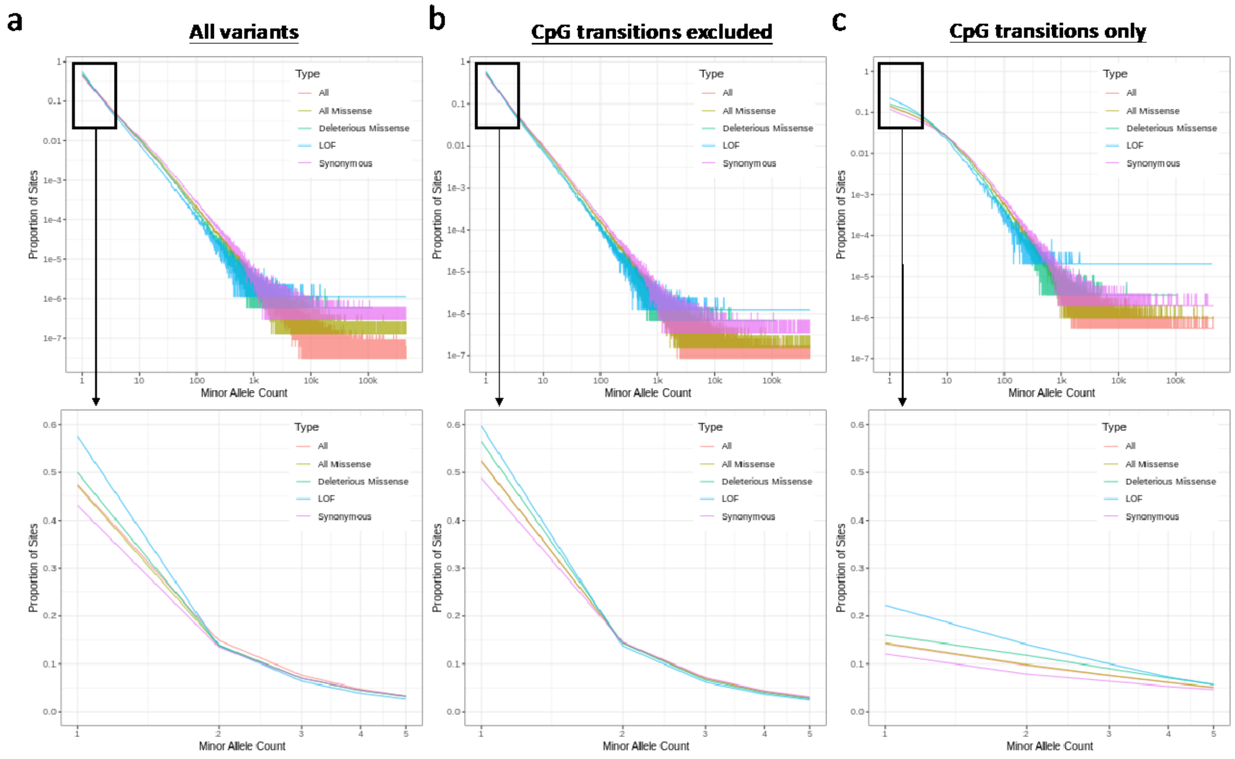

Supplementary Figure 1. Allele frequency spectrum for coding variants identified from exome sequencing of 454,787 individuals in the UK Biobank. a, All 12 million coding variants. **b,** All coding variants except those occurring at CpG transitions. **c,** Only coding variants occurring at CpG transitions. The top plot in each panel shows variants across the full allele frequency spectrum, while the bottom plot shows only variants up to a minor allele count of 5.

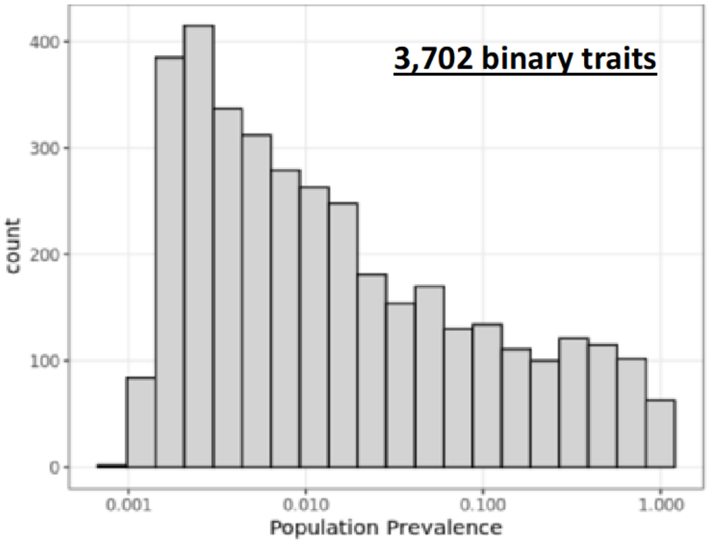

Prevalence	Number of traits
0.1% to 0.5%	1352
0.5% to 1%	519
1% to 5%	869
5% to 10%	267
10% to 20%	214
>20%	481

Supplementary Figure 2. Population prevalence of binary traits tested in individuals of European ancestry from the UK Biobank cohort. We tested 3,702 binary traits with at least 100 cases, of which 1,871 had a population prevalence <1%, 1,136 between 1% and 10%, and 695 >10%. Prevalence was estimated by dividing the number of cases by the total number of individuals with non-missing data, considering only individuals of European ancestry. For female-specific phenotypes (e.g. breast cancer) males were set to missing, and conversely for male-specific phenotypes.

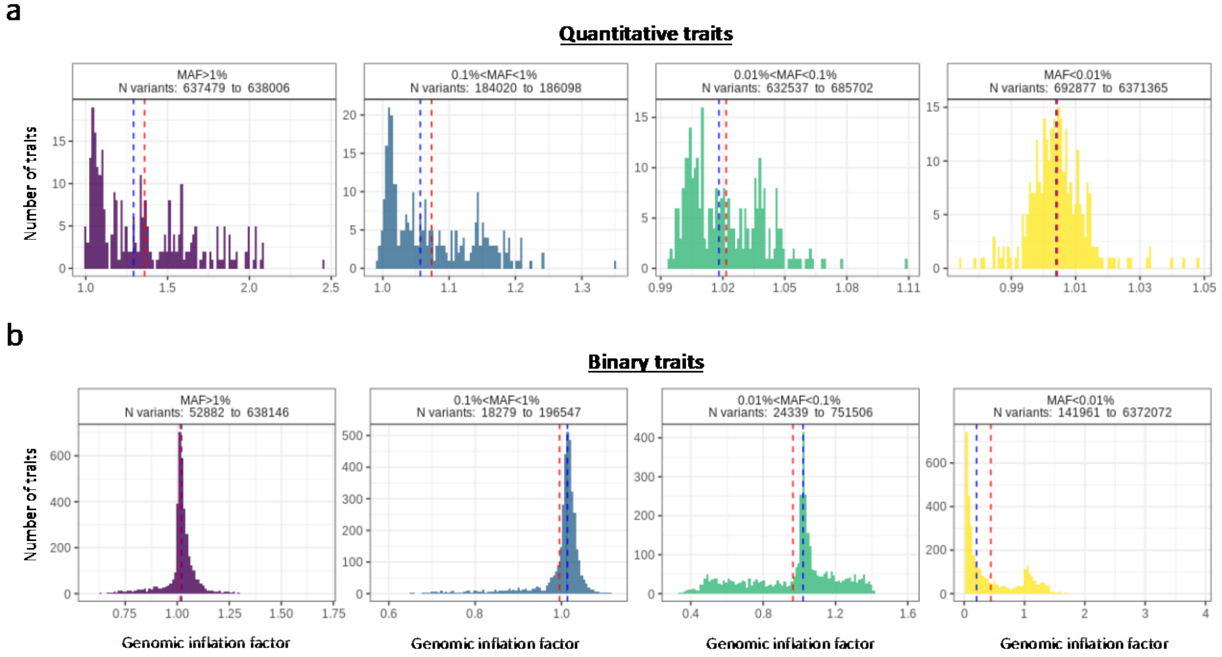

Supplementary Figure 3. Genomic inflation factor (λ) across 3,994 traits tested for association with exome sequencing data from 430,998 individuals of European ancestry from the UK Biobank. a, Results for 292 quantitative traits. **b**, Results for 3,702 binary traits. Genomic inflation factors were calculated separately for variants in four different minor allele frequency (MAF) bins: MAF>1% (purple), 0.1%<MAF<1% (blue), 0.01%<MAF<0.1% (green) and MAF<0.01% (yellow). Red line indicates mean, blue line indicates median. For binary traits, we found that the genomic inflation factor for variants with a MAF<0.01% was <1 for many traits, caused by a large proportion of variants having a minor allele count (MAC) of 0 in affected individuals, as noted previously [15].

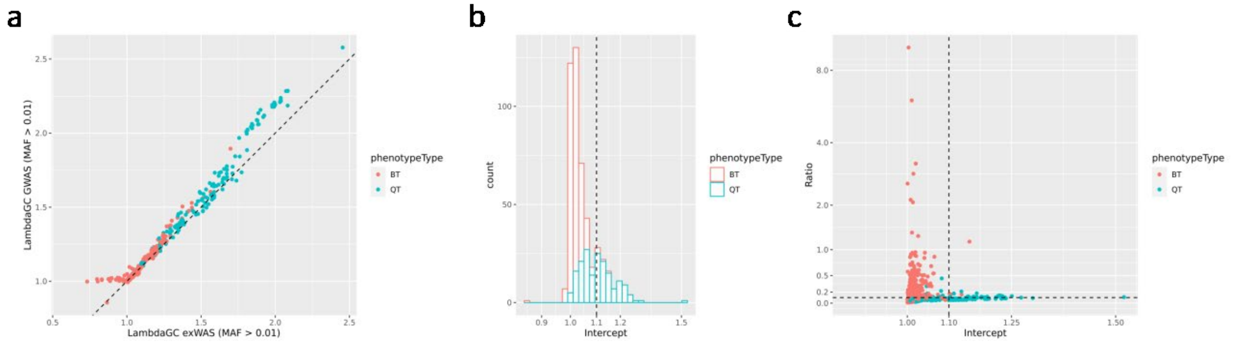

Supplementary Figure 4. Assessment of inflation of association test statistics using genomic inflation factor and LD-score statistics. Results are shown for the 492 traits (314 binary, 178 quantitative) with at least one rare variant association at $P \leq 2.18 \times 10^{-11}$, for which we also ran a GWAS based on TOPMed imputed data. **a,** Comparison of genomic inflation factors (lambda genomic control, GC) for common variants between analysis of exome sequencing data (x-axis, based on about 637,000 variants) and TOPMed imputed data (y-axis, based on about 9.2 million variants). Estimates obtained with imputed data were comparable to those obtained with exome sequencing data, despite the latter including a smaller number of common variants. Given this observation, and the small overlap between common variants from exome sequencing and HapMap 3 variants required to run LD-score regression (LDSC) analyses[16,17], we then used TOPMed imputed data for LDSC analyses. Intercept (**b**) and attenuation ratio (**c**) from LDSC analysis of TOPMed imputed data. We used the more recent LDSC "baseline model" [16] and not the LDSC "original model"[17] because, as described previously[18], we found that the latter produces attenuation ratios that are relatively high (>0.1) when applied to quantitative traits in the UKB cohort. Most binary traits (309 of 314) had an LDSC intercept <1.1, consistent with no substantial impact of population structure or unmodeled relatedness on the results for common variants. Among quantitative traits, 87 (49%) had an LDSC intercept >1.1, but most of these (67 of 87, 77%) had an LDSC attenuation ratio ≤0.1 (y-axis on panel **c**). This is consistent with the relatively

high lambda GC and intercept from LDSC being explained by polygenic effects and not population structure or unmodelled relatedness. Individual values plotted in this figure are provided in **Supplementary Data 1**.

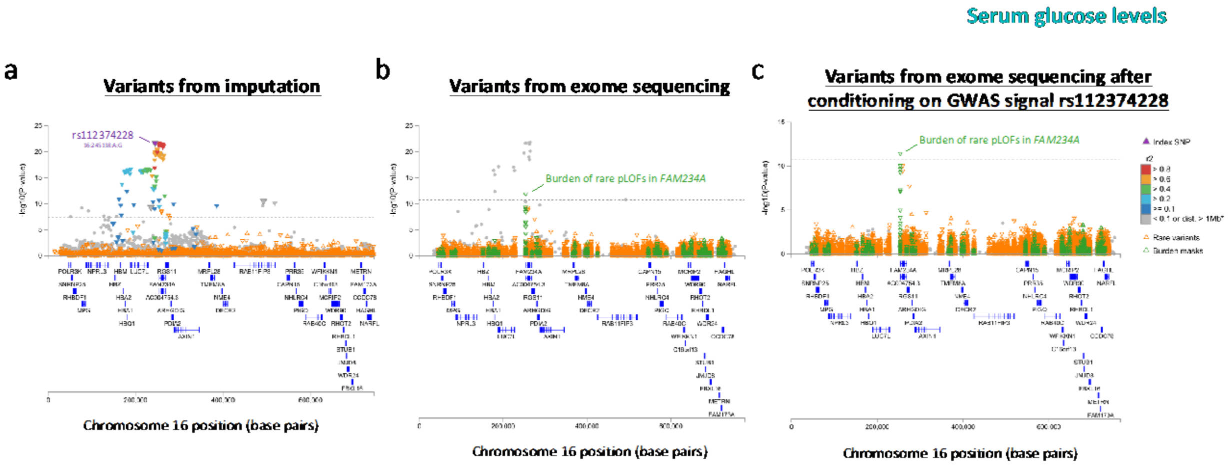

Supplementary Figure 5. Regional association plots for serum glucose levels at the *FAM234A* locus. a, Associations with variants from TOPMed imputed data, showing a single GWAS signal with sentinel variant rs112374228 (MAF=15%). **b,** Associations with variants from exome sequencing, highlighting the most significant burden test between *FAM234A* and serum glucose levels. **c**, Associations with variants from exome sequencing after conditioning on the GWAS sentinel variant rs112374228.

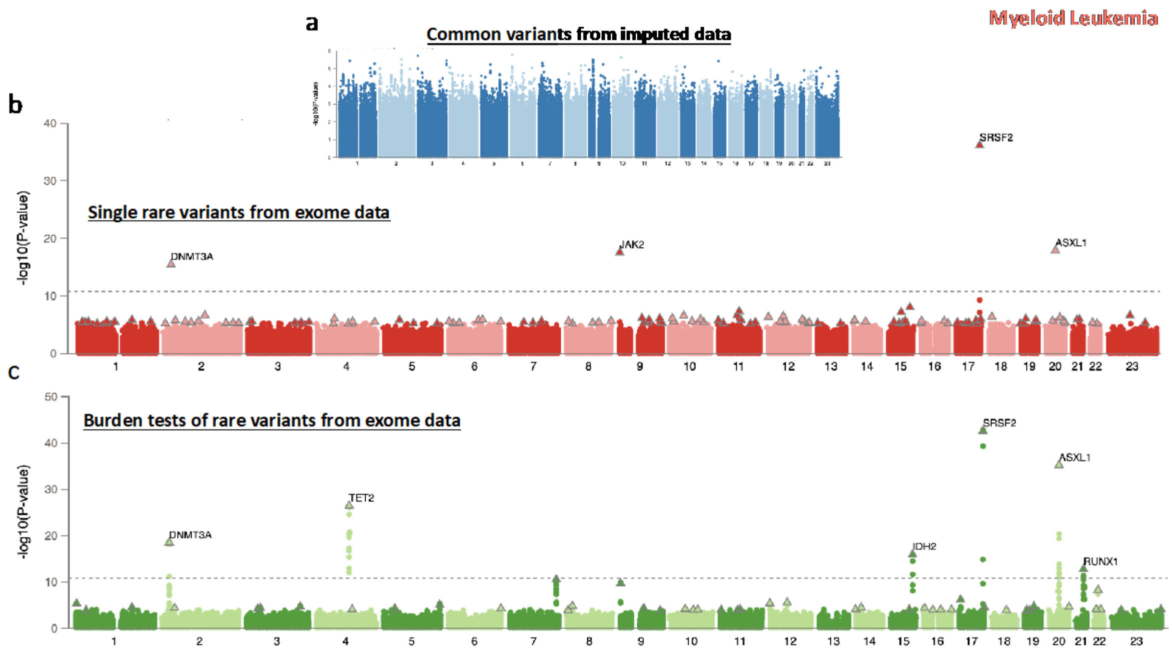

Supplementary Figure 6. The most extreme example of a trait (myeloid leukemia, ICD10 C92) with multiple genes with a rare-variant association but no GWAS signals. a, Associations with common variants from GWAS. **b,** Associations with individual rare variants from exome sequencing. **c,** Burden test associations from exome sequencing. All seven genes with a rare variant association (*DNMT3A*, *TET2*, *JAK2*, *IDH2*, *SRSF2*, *ASXL1* and *RUNX1*) have been described to harbor somatic mutations associated with clonal hematopoiesis of indeterminate significance (CHIP).

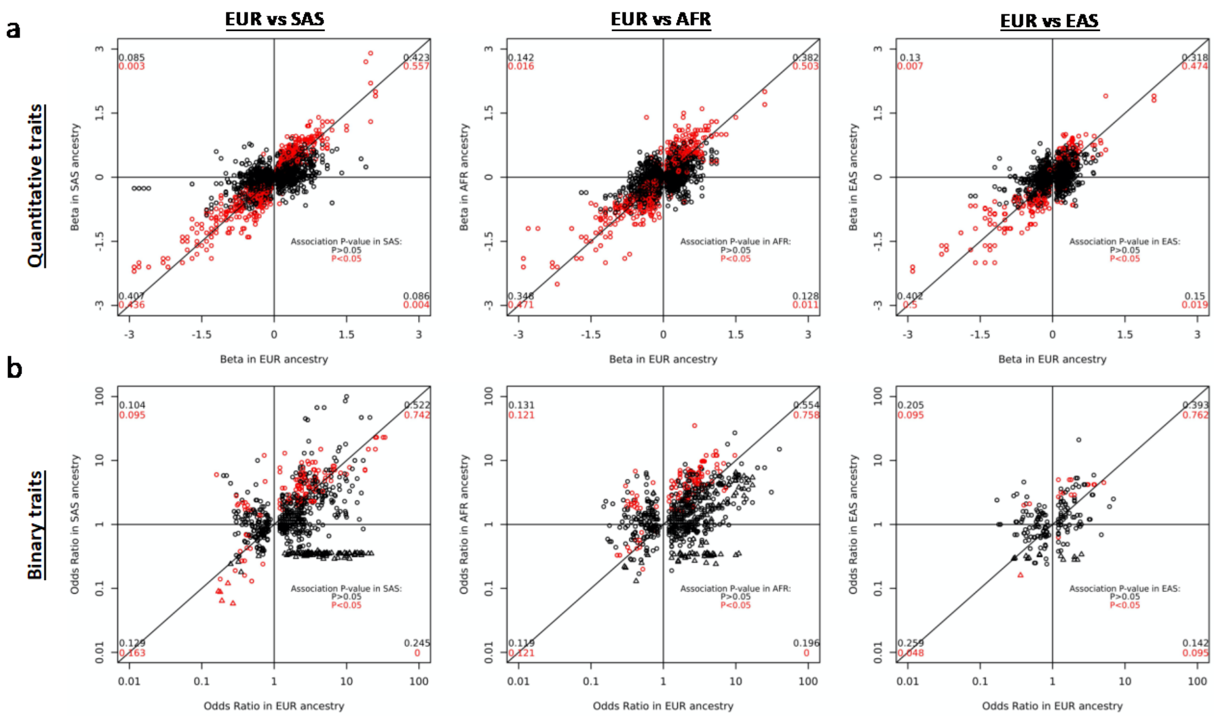

Supplementary Figure 7. Comparison of effect sizes across ancestries for the full 8,865 associations identified in Europeans. For each of the 8,865 associations identified in Europeans (6,498 with a quantitative trait, 2,367 with a binary trait; see **Supplementary Data 2**), we compared the effect size estimated in Europeans with that estimated in individuals of South Asian (SAS), African (AFR) and East Asian (EAS) ancestry, if available. **a,** Of the 6,498 associations with a quantitative trait, 4,321 (83% directionally concordant), 4,178 (73%) and 2,525 (72%) were available in SAS, AFR and EAS, respectively. **b,** Of the 2,367 associations with a binary trait, 1,023 (65% directionally concordant), 996 (67%) and 239 (65%) were available in SAS, AFR and EAS, respectively. Red circles represent associations with $P \leq 0.05$ in the corresponding non-European ancestry. Numbers in the corner of each quadrant represent the proportion of associations in that quadrant, out of the total number of associations in black, and out of the subset a $P \leq 0.05$ in red. Triangles: associations between binary traits and variants for which the minor allele count (MAC) was 0 in affected individuals.

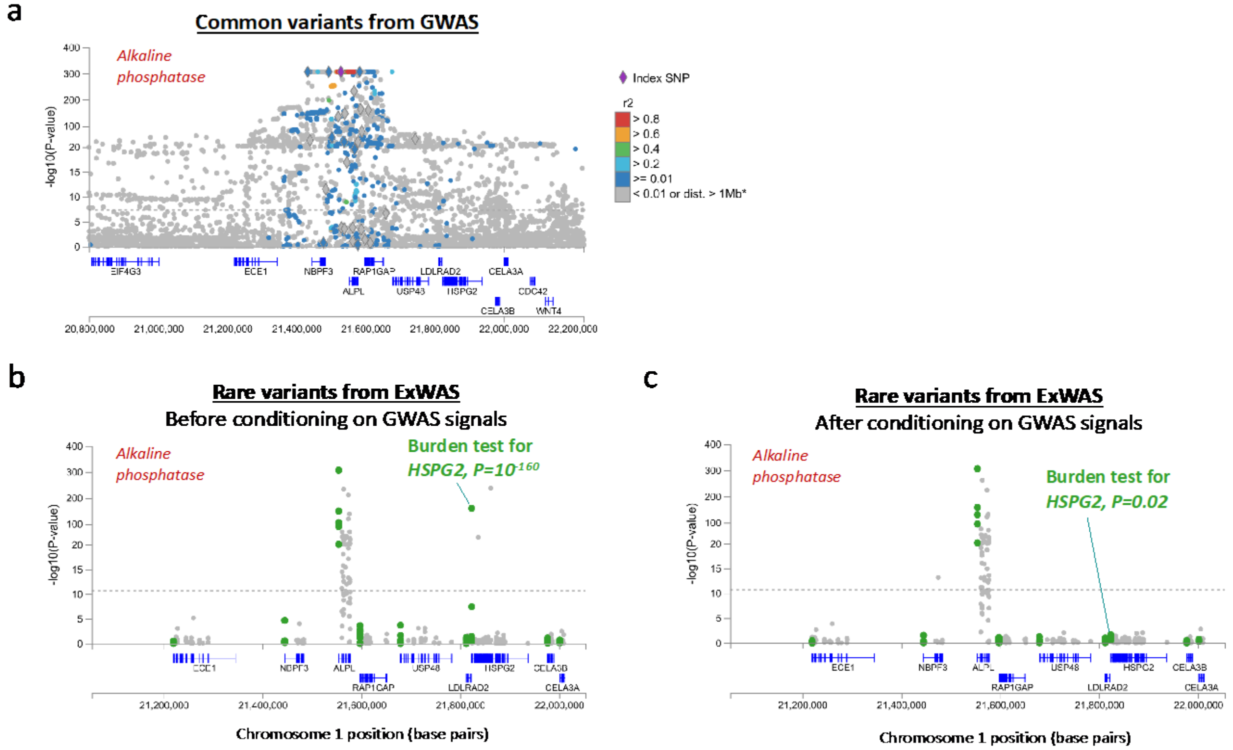

Supplementary Figure 8. Example of a rare variant association (*HSPG2* and alkaline phosphatase) that was significant before but not after conditioning on common variant signals from GWAS. a, Association results for common variants from a GWAS of alkaline phosphatase using TOPMed imputed data. **b,** Association results for rare variants from exome sequencing before conditioning on GWAS signals. **c,** Results for rare variants from exome sequencing after conditioning on GWAS signals. Panels **b** and **c** show results for individual pLOF and deleterious missense variants in grey and burden tests in green.

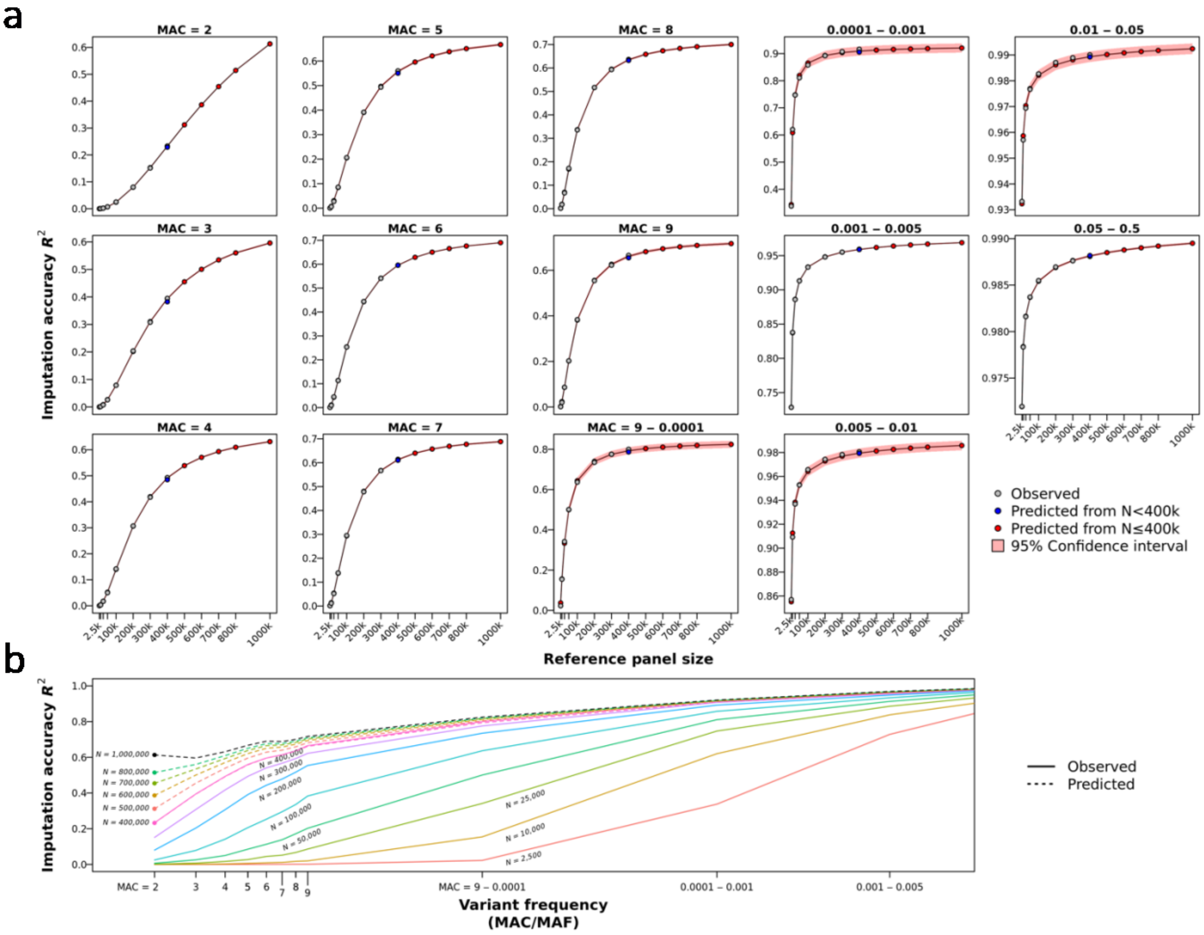

Supplementary Figure 9. Predicted imputation accuracy for variants from exome sequencing as a function of the size of the reference panel using a 3-parameter logistic model. **a,** Each panel shows the imputation accuracy (r^2, y-axis) as a function of the number of individuals included in the reference panel (x-axis), for a given allele frequency bin (estimated in the reference panel). Grey dots show the imputation accuracy that was observed when analyzing reference panels with up to 400,000 individuals. Red dots show the imputation accuracy that was predicted for reference panels with >400,000 individuals, obtained by fitting a 3-parameter logistic curve to results from reference panels with ≤400,000 individuals. The fit from this logistic curve is shown by the solid line, with associated 95% confidence intervals shown in light red. The blue dot is the extrapolated value for a reference panel of 400,000 individuals obtained by fitting the curve using

only reference panels with <400,000 individuals. **b,** Imputation accuracy (r^2, y-axis) is shown as a function of the variant allele frequency (x-axis; minor allele count [MAC] for ultra-rare variants, minor allele frequency [MAF] for variants with MAF>10^{-4}) and the number of individuals (N) included in the reference panel (different lines). Solid lines show the imputation accuracy that was observed when analyzing reference panels with up to 400,000 individuals. Dashed lines show the imputation accuracy that was predicted for reference panels with >400,000 individuals, obtained by fitting a 3-parameter logistic curve to results from reference panels with ≤400,000 individuals.

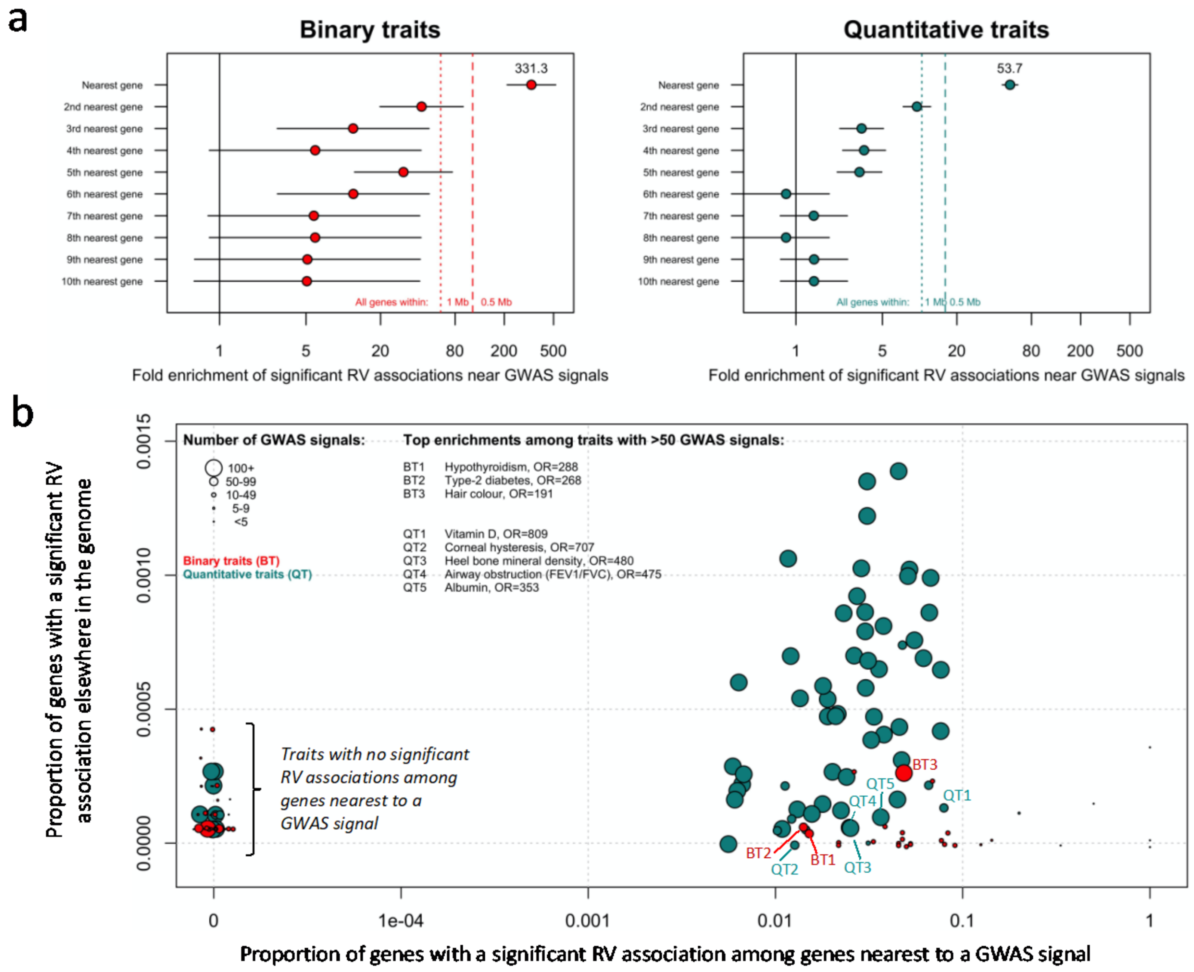

Supplementary Figure 10. Enrichment of rare variant (RV) associations among genes located in GWAS loci. We tested if genes located in GWAS loci were more likely to have significant associations ($P \leq 2.18 \times 10^{-11}$) with a burden of RVs when compared to genes elsewhere in the genome. We considered 13 different gene-sets, from all genes located within 10 Mb of, to only the nearest gene to, the GWAS sentinel variants. **a**, Enrichment of significant associations among genes in GWAS loci was stronger for binary traits (101 included in this analysis) than for quantitative traits (87 included in this analysis). See Supplementary Discussion for caveats. **b**, Comparison of the proportion of significant burden associations ($P \leq 2.18 \times 10^{-11}$) among genes nearest to GWAS sentinel variants (x-axis) and genes located elsewhere in the genome (y-axis).

SUPPLEMENTARY TABLES 1 to 22

Supplementary tables are provided in accompanying Excel file.

Supplementary Table 1. Demographics and clinical characteristics.

Supplementary Table 2. Number of coding variants available in UKB exome sequencing data, gnomAD, TOPMed and UKB TOPMed imputed data.

Supplementary Table 3. Proportion of coding variants in UKB exome sequencing data that were accessible through TOPMed imputation.

Supplementary Table 4. Broad phenotype categories that encompass the 3,994 traits tested for association with rare variants in individuals of European ancestry.

Supplementary Table 5. Number of association tests performed.

Supplementary Table 6. Most significant rare variant-trait association pair for 564 genes with at least one rare variant association with $P \leq 2.18 \times 10^{-11}$ in individuals of European ancestry.

Supplementary Table 7. Risk-lowering associations with disease outcomes in individuals of European ancestry.

Supplementary Table 8. Association between *SLC9A3R2* and blood pressure before and after conditioning on *PKD1* missense variant Arg2200Cys.

Supplementary Table 9. Associations with *SLC9A3R2* and *PIEZO1*, but not *SLC27A3* and *MAP3K15*, were also observed at $P<10^{-7}$ when using TOPMed (instead of exome sequencing) data for assocation analysis (individual variants and burden tests).

Supplementary Table 10. Number of pLOF and deleterious missense variants included in the burden tests for *SLC27A3* and *MAP3K15*, using exome sequencing and TOPMed imputed data.

Supplementary Table 11. Variants for which the direction of effect on a quantitative trait was consistent with a beneficial effect on disease risk.

Supplementary Table 12. Genes for which a variant was associated with both a favorable effect on a quantitative trait and a protective association with a genetically correlated disease.

Supplementary Table 13. Traits with two or more genes with a rare variant association but no GWAS signals.

Supplementary Table 14. Variant allele fraction and association with age for rare variants in genes associated with traits that had no GWAS signals (traits from Supplementary Table 13).

Supplementary Table 15. Gene associations identified in the analysis of non-European but not European ancestries.

Supplementary Table 16. Number of burden associations that remained significant at $P<2.18x10^{-11}$ after adjusting for GWAS signals, stratified by variant class and allele frequency bin.

Supplementary Table 17. Number of burden associations that remained significant at $P<2.18x10^{-11}$ after adjusting for GWAS signals.

Supplementary Table 18. Genes that (i) had a significant burden association after adjusting for GWAS signals; and (ii) were the nearest gene to a GWAS sentinel variant.

Supplementary Table 19. Association between coding and non-coding variation in *HAL* and vitamin D levels and skin-cancer related traits.

Supplementary Table 20. Trait-variant pairs associated with phenotypes derived from brain imaging in individuals of European ancestry at a $P \leq 2.18x10^{-11}$ after adjusting for GWAS signals.

Supplementary Table 21. Trait-variant pairs with a sub-threshold association ($2.18x10^{-11} < P \leq 10^{-7}$) with phenotypes derived from brain imaging in individuals of European ancestry after adjusting for GWAS signals.

Supplementary Table 22. Number of autosomal genes with at least N carriers of rare LOFs (MAF ≤ 1%) in (i) UK Biobank exome sequencing data; (ii) UK Biobank TOPMed imputation; and (iii) expected in 1 and 5 million sequenced individuals.

Supplementary Table 23. Imputation accuracy stratified by reference panel size and minor allele count or minor allele frequency.

SUPPLEMENTARY DATA S1 TO S3

Supplementary datasets S1, S2 and S3 are provided in accompanying Excel file.

Data S1. List of 3,994 traits tested for association with rare variants in individuals of European ancestry from the UK Biobank cohort.

Data S2. Summary statistics for 8,865 associations discovered in the analysis of individuals of European ancestry.

Data S3. Summary statistics for 376 associations discovered in the analysis of individuals of African, East Asian or South Asian ancestries.

Data S4. Accession numbers for summary statistics available through the GWAS catalog.

References

1 Malik, I. A. *et al.* Sepsis and Acute Myeloid Leukemia: A Population-Level Study of Comparative Outcomes of Patients Discharged From Texas Hospitals. *Clin Lymphoma Myeloma Leuk* **17**, e27-e32, doi:10.1016/j.clml.2017.07.009 (2017).

2 Lai, T. S. *et al.* Risk of developing severe sepsis after acute kidney injury: a population-based cohort study. *Crit Care* **17**, R231, doi:10.1186/cc13054 (2013).

3 Wu, M. C. *et al.* Rare-variant association testing for sequencing data with the sequence kernel association test. *Am J Hum Genet* **89**, 82-93, doi:10.1016/j.ajhg.2011.05.029 (2011).

4 Lee, S. *et al.* Optimal unified approach for rare-variant association testing with application to small-sample case-control whole-exome sequencing studies. *Am J Hum Genet* **91**, 224-237, doi:10.1016/j.ajhg.2012.06.007 (2012).

5 Yang, J. *et al.* Conditional and joint multiple-SNP analysis of GWAS summary statistics identifies additional variants influencing complex traits. *Nature genetics* **44**, 369-375, S361-363, doi:10.1038/ng.2213 (2012).

6 Hindy, G. *et al.* Rare coding variants in 35 genes associate with circulating lipid levels – a multi-ancestry analysis of 170,000 exomes. *bioRxiv*, 2020.2012.2022.423783, doi:10.1101/2020.12.22.423783 (2021).

7 Zuk, O. *et al.* Searching for missing heritability: designing rare variant association studies. *Proc Natl Acad Sci U S A* **111**, E455-464, doi:10.1073/pnas.1322563111 (2014).

8 Finucane, H. K. *et al.* Partitioning heritability by functional annotation using genome-wide association summary statistics. *Nature genetics* **47**, 1228-1235, doi:10.1038/ng.3404 (2015).

9 Edwards, S. L., Beesley, J., French, J. D. & Dunning, A. M. Beyond GWASs: illuminating the dark road from association to function. *Am J Hum Genet* **93**, 779-797, doi:10.1016/j.ajhg.2013.10.012 (2013).

10 Mochel, F. *et al.* Adult polyglucosan body disease: Natural History and Key Magnetic Resonance Imaging Findings. *Ann Neurol* **72**, 433-441, doi:10.1002/ana.23598 (2012).

11 Nguyen, T. T. *et al.* PLEKHG3 enhances polarized cell migration by activating actin filaments at the cell front. *Proc Natl Acad Sci U S A* **113**, 10091-10096, doi:10.1073/pnas.1604720113 (2016).

12 Cirulli, E. T. *et al.* Genome-wide rare variant analysis for thousands of phenotypes in over 70,000 exomes from two cohorts. *Nat Commun* **11**, 542, doi:10.1038/s41467-020-14288-y (2020).

13 McMahon, F. J. *et al.* Meta-analysis of genome-wide association data identifies a risk locus for major mood disorders on 3p21.1. *Nature genetics* **42**, 128-131, doi:10.1038/ng.523 (2010).

14 Lee, J. J. *et al.* Gene discovery and polygenic prediction from a genome-wide association study of educational attainment in 1.1 million individuals. *Nature genetics* **50**, 1112-1121, doi:10.1038/s41588-018-0147-3 (2018).

15 Kosmicki, J. A. *et al.* Pan-ancestry exome-wide association analyses of COVID-19 outcomes in 586,157 individuals. *Am J Hum Genet* **108**, 1350-1355, doi:10.1016/j.ajhg.2021.05.017 (2021).

16 Gazal, S. *et al.* Linkage disequilibrium-dependent architecture of human complex traits shows action of negative selection. *Nature genetics* **49**, 1421-1427, doi:10.1038/ng.3954 (2017).
17 Bulik-Sullivan, B. K. *et al.* LD Score regression distinguishes confounding from polygenicity in genome-wide association studies. *Nature genetics* **47**, 291-295, doi:10.1038/ng.3211 (2015).
18 Loh, P. R., Kichaev, G., Gazal, S., Schoech, A. P. & Price, A. L. Mixed-model association for biobank-scale datasets. *Nature genetics* **50**, 906-908, doi:10.1038/s41588-018-0144-6 (2018).
