# Supplementary material for: Exome sequencing and analysis of 454,787 UK Biobank participants
Source: Nature. 2021 Oct 18;599(7886):628–34. doi: 10.1038/s41586-021-04103-z (PMC8596853; doi:10.1038/s41586-021-04103-z)
Supplement: Supplementary file 2 — Reporting Summary [file 41586_2021_4103_MOESM2_ESM.pdf]

nature portfolio

Corresponding author(s): Manuel Ferreira and Goncalo Abecasis

Last updated by author(s): Sep 9, 2021

Reporting Summary

Nature Portfolio wishes to improve the reproducibility of the work that we publish. This form provides structure for consistency and transparency in reporting. For further information on Nature Portfolio policies, see our Editorial Policies and the Editorial Policy Checklist.

Statistics

For all statistical analyses, confirm that the following items are present in the figure legend, table legend, main text, or Methods section.

n/a	Confirmed	
☐	☒	The exact sample size (*n*) for each experimental group/condition, given as a discrete number and unit of measurement
☐	☒	A statement on whether measurements were taken from distinct samples or whether the same sample was measured repeatedly
☐	☒	The statistical test(s) used AND whether they are one- or two-sided<br>*Only common tests should be described solely by name; describe more complex techniques in the Methods section.*
☐	☒	A description of all covariates tested
☐	☒	A description of any assumptions or corrections, such as tests of normality and adjustment for multiple comparisons
☐	☒	A full description of the statistical parameters including central tendency (e.g. means) or other basic estimates (e.g. regression coefficient) AND variation (e.g. standard deviation) or associated estimates of uncertainty (e.g. confidence intervals)
☐	☒	For null hypothesis testing, the test statistic (e.g. *F*, *t*, *r*) with confidence intervals, effect sizes, degrees of freedom and *P* value noted<br>*Give P values as exact values whenever suitable.*
☒	☐	For Bayesian analysis, information on the choice of priors and Markov chain Monte Carlo settings
☐	☒	For hierarchical and complex designs, identification of the appropriate level for tests and full reporting of outcomes
☐	☒	Estimates of effect sizes (e.g. Cohen's *d*, Pearson's *r*), indicating how they were calculated

Our web collection on statistics for biologists contains articles on many of the points above.

Software and code

Policy information about availability of computer code

Data collection	No software was used for data collection.
Data analysis	The association analysis package used to perform all genetic associations is available at https://github.com/rgcgithub/regenie. GCTA v1.91.7 was used for approximate conditional analysis. LDSC v1.0.1 was used LD score regression. with SHAPEIT4.2.0 was used for phasing of SNP array data. Imputation was completed with IMPUTE5.

For manuscripts utilizing custom algorithms or software that are central to the research but not yet described in published literature, software must be made available to editors and reviewers. We strongly encourage code deposition in a community repository (e.g. GitHub). See the Nature Portfolio guidelines for submitting code & software for further information.

Data

Policy information about availability of data

All manuscripts must include a data availability statement. This statement should provide the following information, where applicable:

- Accession codes, unique identifiers, or web links for publicly available datasets
- A description of any restrictions on data availability
- For clinical datasets or third party data, please ensure that the statement adheres to our policy

Individual-level sequence data have been deposited with UK Biobank and will be freely available to approved researchers, as done with other genetic datasets to date. Individual-level phenotype data are already available to approved researchers for the surveys and health-record datasets from which all our traits are derived. Instructions for access to UK Biobank data is available at https://www.ukbiobank.ac.uk/enable-your-research. Full details for the trait associations with rare variants described in this study are provided in Data S2 and S3. The HapMap3 reference panel was downloaded from ftp://ftp.ncbi.nlm.nih.gov/hapmap/. GnomAD v3.1 VCFs were obtained from https://gnomad.broadinstitute.org/downloads. VCFs for TOPMED Freeze 8 were obtained from dbGaP as described in https://

topmed.nhlbi.nih.gov/topmed-whole-genome-sequencing-methods-freeze-8. LD scores from 1000 Genomes Project were downloaded from https://data.broadinstitute.org/alkesgroup/LDSCORE/.

Field-specific reporting

Please select the one below that is the best fit for your research. If you are not sure, read the appropriate sections before making your selection.

☒ Life sciences ☐ Behavioural & social sciences ☐ Ecological, evolutionary & environmental sciences

For a reference copy of the document with all sections, see nature.com/documents/nr-reporting-summary-flat.pdf

Life sciences study design

All studies must disclose on these points even when the disclosure is negative.

Sample size	Sample size was not predetermined. Association analyses were restricted to the intersection of samples with both exome sequence and array genotypes available after QC. See methods section "Exome sequencing" for details on QC performed. All samples that pass genotype QC and with non-missing phenotype data were included in association analyses. We performed power calculations (Extended data figure 4) that suggest we are well-powered to detect genetic associations under a variety of scenarios, although there may be some traits for which we did not have adequate sample size.
Data exclusions	Phenotype selection and QC was performed as described in methods section "Health- and behavior-related phenotypes." Variant level QC was performed as described in methods section "Exome sequencing." Variants with minor allele count less than five were excluded from association testing. The minor allele count threshold was pre-determined based on extensive simulations performed with REGENIE. See https://www.nature.com/articles/s41588-021-00870-7 for additional details.
Replication	Replication was attempted for all significant variant-trait associations available for follow-up in the DiscovEHR study. 81% of associations available and powered for replication were confirmed.
Randomization	Randomization was not required for the analyses completed in this study. To control for confounding, we performed association analysis with the following covariates included in the regression model: age, age-squared, sex, age-x-sex, 10 ancestry-informative principal components, six exome sequence batch indicator variables, and 20 principal components derived from exome variants with a MAF between 2.6x10-5 and 1%.
Blinding	Blinding was not required for the analyses completed in this study. Participant recruitment and phenotype collection were obtained without prior knowledge of sample genotypes. Association analyses were performed with all available samples, without any filtering based on sample genotypes.

Reporting for specific materials, systems and methods

We require information from authors about some types of materials, experimental systems and methods used in many studies. Here, indicate whether each material, system or method listed is relevant to your study. If you are not sure if a list item applies to your research, read the appropriate section before selecting a response.

Materials & experimental systems

n/a	Involved in the study	
☒	☐	Antibodies
☒	☐	Eukaryotic cell lines
☒	☐	Palaeontology and archaeology
☒	☐	Animals and other organisms
☐	☒	Human research participants
☒	☐	Clinical data
☒	☐	Dual use research of concern

Methods

n/a	Involved in the study	
☒	☐	ChIP-seq
☒	☐	Flow cytometry
☒	☐	MRI-based neuroimaging

Human research participants

Policy information about studies involving human research participants

Population characteristics	The UK Biobank is a prospective cohort study previously described in detail by Bycroft et al, Nature 2018 (https://www.nature.com/articles/s41586-018-0579-z). Briefly, 94.7% of sequenced participants are of European ancestry, 54.2% are female, the average age at assessment is 58, and the mean BMI is 26. 45% of participants report a history of smoking, and each participant reports 8 inpatient ICD10 3D codes, on average. See supplementary table 1 for additional details.
Recruitment	Please see Bycroft et al, Nature 2018.
Ethics oversight	Ethical approval for the UK Biobank was previously obtained from the North West Centre for Research Ethics Committee (11/NW/0382). The work described herein was approved by UK Biobank under application number 26041. Approval for

DiscovEHR analyses was provided by the Geisinger Health System Institutional Review Board under project number 2006-0258. Informed consent was obtained for all study participants.

Note that full information on the approval of the study protocol must also be provided in the manuscript.
